# Operations management solutions to improve ED patient flows: evidence from the Italian NHS

**DOI:** 10.1186/s12913-022-08339-x

**Published:** 2022-07-30

**Authors:** Marta Marsilio, Eugenia Tomas Roldan, Luca Salmasi, Stefano Villa

**Affiliations:** 1 Department of Economics, Management and Quantitative Methods (DEMM), Università degli Studi di Milano, Milano, Italy; 2 CERISMAS (Research Centre in Health Care Management), Università Cattolica del Sacro Cuore, Milano, Italy; 3 Department of Economics and Finance, Università Cattolica del Sacro Cuore, Roma, Italy; 4 Department of Management, Università Cattolica del Sacro Cuore, Milano, Italy

**Keywords:** Emergency department, Length of stay (LOS), Patient flow logistics, Healthcare operations management

## Abstract

**Background:**

Overcrowding occurs when the identified need for emergency services outweighs the available resources in the emergency department (ED). Literature shows that ED overcrowding impacts the overall quality of the entire hospital production system, as confirmed by the recent COVID-19 pandemic. This study aims to identify the most relevant variables that cause ED overcrowding using the input-process-output model with the aim of providing managers and policy makers with useful hints for how to effectively redesign ED operations.

**Methods:**

A mixed-method approach is used, blending qualitative inquiry with quantitative investigation in order to: i) identifying and operationalizing the main components of the model that can be addressed by hospital operation management teams and ii) testing and measuring how these components can influence ED LOS.

**Results:**

With a dashboard of indicators developed following the input-process-output model, the analysis identifies the most significant variables that have an impact on ED overcrowding: the type (age and complexity) and volume of patients (input), the actual ED structural capacity (in terms of both people and technology) and the ED physician-to-nurse ratio (process), and the hospital discharging process (output).

**Conclusions:**

The present paper represents an original contribution regarding two different aspects. First, this study combines different research methodologies with the aim of capturing relevant information that by relying on just one research method, may otherwise be missed. Second, this study adopts a hospitalwide approach, adding to our understanding of ED overcrowding, which has thus far focused mainly on single aspects of ED operations.

## Introduction

In recent years, both practitioners and academics have recognized the strategic role of operations management in healthcare delivery organizations. A better management of the flows of patients and materials throughout different healthcare production units is critical in dealing with the current challenges of healthcare systems and improving the overall quality of the care provided.

In this context, the emergency department (ED) plays a relevant role as, together with the outpatient departments, is one of the two entry points to the hospital production system [1].

Even the case-based evidence drawn from the recent pandemic shows that the ED has been crucial in the management of COVID-19 patients [2, 3]: well-prepared and well-functioning EDs have achieved better results in terms of the timelines, responsiveness, and capability of separating patient flows [4]. In the last decade, EDs worldwide have faced the challenges of cost containment, excessive waiting times, and overcrowding [5, 6]. The literature provides different possible definitions and interpretations of this concept. In this paper, we adopt the interpretation of the American College of Emergency Physicians, which defines overcrowding as *“a situation in which the identified need for emergency services outstrips available resources in the ED. This situation occurs in hospital EDs when there are more patients than staffed ED treatment beds and wait times exceed a reasonable period”* [7] (p.174)*.* ED overcrowding has a relevant impact on several aspects of the overall quality of the care provided by hospitals [8–11], such as: the ability to provide critical services to patients suffering from actual medical emergencies in a timely manner; the working climate since overcrowding creates frustration among ED staff (both nurses and medical doctors); patient safety and satisfaction; the treatment of patients in inappropriate infrastructure settings; and patients’ clinical outcomes, including higher mortality rates, errors, adverse events and increased morbidity.

By applying operations management principles to unscheduled emergency patient flow, Asplin et al. [7] developed one of the most cited conceptual models of ED crowding. This model identifies the three main components of ED crowding: input, throughput/process and output. The input-process-output model is quite diffuse for the analysis of health production processes [12–14]. Considering ED flow, the input component includes any condition, event, or system feature that contributes to the demand for ED services. The throughput (process) component identifies patient lenght of stay (LOS) in the ED as a potential contributing factor to ED crowding. Thus, ED care processes must be accurately analysed, managed and properly (re)designed to improve their efficiency and effectiveness. Specifically, in the ED, patient flows can be broken up into four different phases: i) admission and waiting for the medical visit, ii) diagnostic and treatment time, iii) transfer to and stay in the observation unit, and iv) waiting for a hospital bed (so-called boarding time). Finally, the output phase deals with all the coordination issues between the ED and all other possible downstream settings where the patient may end up, like (i) a hospital inpatient bed, (ii) ambulatory care, (iii) his or her home, or (iv) intermediate care (e.g., nursing homes or rehabilitation centres).

This conceptual model provides a general framework with which to study the causes and consequences of ED crowding, but it is too broad and vague to be applied as an ED operations management tool, requiring the development of measurements for each component [7].

To date, the literature has focused mainly on a few specific variables of the three components of this framework, providing piecemeal solutions to ED crowding rather than integrated solutions [15]. According to a recent systematic literature review, the majority of studies (60%) that have reported on potential solutions to ED crowding have focused on expediting patients' throughput within the ED; very few studies have addressed the issue of dividing patients by age, level of acuity or triage code; and none have aimed specifically at improving staffing issues. This situation suggests a mismatch between the proven or accepted causes of crowding and the solutions developed and implemented to address the problem.

To fill this gap, this paper aims to achieve two different goals: (i) the operationalization of the input-process-output model through the development of an integrated, balanced dashboard of indicators that are useful for measuring the performance of emergency patient flow management and (ii) the understanding of the most relevant explicatory variables of ED LOS to provide managers and policy makers with an evidence-based road map for the redesign of ED patient flows.

In particular, the study adopts an operations management hospitalwide perspective, focusing on unscheduled patient flow management from the very first hospital access of the patient to final discharge, with the goal of investigating the whole patient journey throughout the different hospital production units to capture the interdependencies between the ED and units involved (wards, outpatient platforms, operating rooms and diagnostics). In the literature background” section, the main evidence on the variables explaining ED LOS within each component are traced. Then, this paper adopts a mixed-method approach with a three-step sequential exploratory design. Qualitative analysis informs the identification and measurement of the possible explicatory variables of ED crowding for each component through focus groups with ED operation managers of the Italian National Health System (INHS); then, the relevance of each variable is tested with a regression model to identify the most important causes of ED crowding from each model component. Then, the results are discussed in a final focus group with the same ED operation management experts. Finally, this work draws some implications for both policy makers and hospital managers.

## Literature background

Assuming a hospital perspective for the analysis of ED LOS through the input-process-outcome model, crowding in the ED can occur due to the type and volume of patients waiting to be seen (input), delays in assessing or treating those patients already in the ED (process), or impediments to patients leaving the ED once their treatment has been completed (output) [15]. In previous years, several works have addressed and empirically tested the effect of specific variables on ED crowding for each component.

Regarding input, some studies have outlined the relevance of patient characteristics as important explicatory variables of ED crowding. In particular, different authors have focused on patient age as a possible explanatory variable of ED overcrowding. George et al*.* [16] examined patient age as a possible cause of ED overcrowding and longer wait times and found that elderly people had a rate of admission five times higher than that of patients aged less than 30 years. Many patients who live in residential homes or are enrolled in home care programs suffer from severe comorbidities and are sent to the ED for care [16]. Once in the ED, elderly individuals require more critical care, more physician time and more examinations [17]. Increased ED presentations by elderly individuals, as a factor contributing to crowding, was also found by a Canadian retrospective cohort study [18]. Likewise, a Japanese study that undertook a cross-sectional analysis of all adult ED presentations at one ED concluded that older people in the ED had a significant negative impact on ED crowding [19]. Furthermore, Kawano et al. [19] reported that crowding worsened as the mean age of patients in the ED increased.

Second, patients arriving in the ED are assigned a priority code based on the severity of the disease; this assessment process is known as “triage”. The role of the ED should be focused on providing care to patients with sudden deterioration or sudden and potentially severe manifestations of an acute illness or injury. Nonetheless, in reality, a larger variety of patients seek care in the ED [20]*.* However, few studies have analysed the impact of patient codes on ED operations and crowding.

Finally, in the analysis of the input component, it is important to consider that EDs play an important role as a “safety net” for vulnerable populations in the community. We refer here to categories such as uninsured or underinsured patients, homeless patients, and psychiatric patients for whom an ED is often the only place where they can receive assistance and care [11, 21].

In conclusion, the latest contributions outline that the characteristics of patient arrivals, mainly age, complexity, and level of urgency, have a relevant impact on ED crowding [15].

For the throughput component, ED processes are plagued by several problems. Some studies have shown that shortages of nurses, junior medical staff and specialty doctors have a strong impact on crowding [22]. Particularly, ED nursing staff shortages as a cause of ED crowding were highlighted in exploratory fieldwork undertaken with 158 ED directors in Canada [23] and in one American study that surveyed 210 ED directors [24]. Using staff differently and hiring additional profiles were found to help alleviate ED overcrowding. For instance, hospitals can hire scribes to handle documentation tasks since it is estimated that emergency physicians spend between 90 and 120 min per 8 h on documentation. Additionally, the use of mental health nurses to provide special support, social workers to help facilitate timely discharge, and patient flow coordinators to coordinate admissions, diagnostic results, and so forth can reduce overcrowding problems in the ED [10, 25]. The introduction of new clinical roles (for example, a new nursing coordinator for hospital medical wards on afternoon shifts, a new target nursing coordinator role in the ED, and a new nursing coordinator role for patient flows in ED) have helped improve patient flow from the ED to hospital inpatient departments [26]. It is important to specify that in these latter studies, the scenarios depicted imply the constitution of new positions that obviously do not have a negligible economic impact. In contrast, no studies have analysed the impact of a skill mix change on ED operations.

Numerous other factors**,** such as poor physical design and a shortage of physical space, equipment and computers, difficulties in accessing medical notes, tests, results and ancillary services, and time spent discharging or arranging follow-up appointments, are all thought to contribute to ED crowding [27]. For example, one study [28] proved that reducing the time needed for laboratory response by 50% had an impact on the overall ED LOS by approximately 15%. The use of observation units (OU) can increase patient safety and satisfaction while decreasing unnecessary inpatient admissions and improving financial performance for both emergency departments and the hospitals in which they operate [29]. One French study showed how the setup of alternative structures, such as primary care units inside or near the ED, seems to respond appropriately to the growing demand of nonurgent patients and their willingness to accept reorientation to an alternative healthcare structure [30]. The setup of performance monitoring systems brings about different dimensions of improvements, such as (i) triage wait time, (ii) number of admitted patients waiting more than 8 h for a hospital bed, (iii) increase in patients discharged from hospitals before the target of noon and (iv) decrease in ED LOS [31]. The adoption of the improvement of patient flow techniques in the emergency department, such as visual stream mapping, can improve the efficiency of services and reduce waste (wait times) [32].

Finally, several studies have focused on the output component, indicating the inability to transfer patients to inpatient beds as the main cause of ED patient flow problems. Several studies have shown a direct link between innovation in discharge management practice and ED wait time. For example, Villa and colleagues [33] found a correlation between the ED LOS and proportion of patients discharged by noon. This link between patients’ discharging process and ED overcrowding has also been proven by other studies. For example, Beck and colleagues [34] showed how the use of a bedside discharge process checklist with an afternoon planning huddle increased the number of discharges before noon and decreased ED wait time. Moreover, Wertheimer and colleagues [35] highlighted how the implementation of an afternoon meeting to address discharge barriers for next-day discharge was associated with a more-than-triple increase in the number of next-day discharges before noon and a decrease in ED LOS.

In general, the better management of patients in the ward alleviates the pressure placed on the ED. For example, the removal of urethral catheters at midnight prior to discharge, compared to at 6 am the day of discharge, was found to be associated with a 163.6% decrease in the number of patients discharged later in the same day [36], while the centralization of the acceptance authority for multiple medical services with one hospitalist, along with rounds in critical care areas to update shared capacity information, resulted in a decrease in the number of hours of ED diversion and a decrease in ED LOS for patients admitted to critical care beds [37]. Changes to discharge processes and connections (handover) to aged care facilities were introduced to move patients more efficiently through the ED and hospital [25].

## Methods

The literature offers interesting empirical evidence on ED crowding. Despite the growing awareness that ED problems are caused by drawbacks in the overall logistics system for hospital patient flows [11, 27], the literature still focuses mainly on a few specific variables of the three components of the input-throughput-output model. The present work aims to fill this gap by i) identifying and operationalizing the main components of the model that can be addressed by hospital operation management teams and ii) testing and measuring how these components can influence ED LOS.

To achieve these goals, this study adopts a mixed-method (MM) approach [38], which increases the breadth and range of the study findings, capturing relevant information that may be missed by relying exclusively on a single research method and which, in general, enhances and strengthens the research results [39]. Specifically, to develop and test an input-process-output hospital ED operation management framework, a three-phase sequential exploratory design is developed [40].

### Phase 1—qualitative methods: framework development

In the first phase, a qualitative approach is used to identify and operationalize the key drivers that can be addressed by hospital operation management teams to deal with the ED LOS issue. Specifically, the authors organize two focus groups with professionals from 10 (ten) Italian hospitals to derive those variables that can be identified as the key drivers that must be addressed when dealing with ED operation management inside the hospital. The focus groups last three hours each, taking place in May and October 2019.

Participants are employees of Italian hospitals belonging to a professional network coordinated by a consortium of universities that promote the adoption of operations management concepts to patient flow in the INHS through research activities and executive education programs. The INHS is a publicly funded system that guarantees universal coverage; it is structured along three tiers: the central government at the top, 21 regional governments in the middle, and approximately 200 local health authorities (LHA) with their own hospitals and other healthcare facilities at the bottom. Each citizen is assigned to an LHA but can freely choose to receive care from any other LHA, public independent trusts (nearly 100) or all private hospitals accredited by the National Health System (NHS). The boards of network hospitals are invited to select a team of professionals involved in ED operations management who have taken part in the focus groups. For the hospital board, participation in this research program is seen as valuable, as the information produced during the different research stages can provide useful insights that can be put into practice by participant teams in their own ED hospital settings.

The main characteristics of the ten hospitals are summarized in Table 1.

**Table 1:** Main characteristics of the ten hospitals included in the study

**Hospital**	**Ownership**	**Bed size**	**ED type**^a^
A	Private Nonprofit Teaching Hospital	1,154	Level II
B	Public–Private Partnership Non-Teaching Hospital	615	Level II
C	Public Hospital within an LHA	230	General
D	Public Hospital within an LHA	618	Level II
E	Public Hospital within an LHA	355	Level II
F	Public Hospital within an LHA	238	General
G	Public Independent Teaching Hospital	547	Level II
H	Public Independent Teaching Hospital	1,121	Level II
I	Public Hospital within an LHA	184	Level I
L	Public Independent Teaching Hospital	271	Level II

^a^Within the Italian NHS, there are three different levels of hospitals emergency units: (i) level II ED for the most complicated cases, with the presence of the highly specialized centres such as a trauma centre, organ transplants and neurosurgery, and catchment area between 600,000 and 1,200,000 inhabitants, with a number of yearly access instances higher than 70,000; (ii) level I emergency unit capable of treating all clinical conditions, and catchment area of 150,000/300,000 inhabitants and approximately 45,000 yearly access instances; and (iii) general emergency hospitals that guarantee emergency access but not high specialization, and catchment area of 80,000/150,000 inhabitants and no more than 20,000 access instances per year

Table 2 below illustrates, for each hospital, the role of the professionals identified as taking part in this ED operations redesign; these professionals have direct strategic responsibility over hospital operations processes, including ED settings. The different compositions of hospital teams reflect the identification of different roles and responsibilities linked to ED operations management activities in each hospital.

**Table 2:** Role of professionals involved in the focus groups

Hospital	N	Roles of professionals
A	3	Chief Medical Officer, Chief Operating Officer, Chief Nursing Officer
B	4	Chief Medical Officer, Hospital Director, Chief Nursing Officer, Nurse Coordinator
C	2	Chief Medical Officer, Chief Nursing Officer
D	6	Chief Medical Officer, Medical Director, Chief Operating Officer, Chief of Lean Team, 2 Members of Lean Team
E	4	Chief Medical Officer, Chief Medical Officer, Chief of Organizational Innovation, Chief of ICT
F	4	Chief Nursing Officer, Chief of Financial Office, Chief Quality Assurance Officer, Medical Director
G	1	ED Medical Director
H	6	Chief Medical Officer, Chief of Financial Office, Project Manager, Chief Nursing Officer, 1 Surgeon, 1 Anaesthetist
I	2	Chief Medical Office, Chief Nursing Officer
L	1	Chief of Organizational Innovation
***Tot***	***33***	

During the first round, the authors present the available literature on the input-process-output model discussed in the background session to the professionals and asked them to identify i) the variable for each input-process-output component that can be addressed by the ED OM team to redesign ED operations and ii) the possible indicators to be used to operationalize each variable, considering the specific data/information to be gathered for calculation. Participants use action priority maps to identify the components and a specific, measurable, achievable, reasonable, and time-bound (SMART) classification to rank the indicators that emerged for each component.

The results of this first round are presented by the authors during the second focus group, aiming to let the professionals revise, elaborate, or validate their previous responses to set the framework to be tested with regression analysis. Participants are asked to offer their own interpretations of those variables/indicators that are not recognized as significant during the first round and to confirm the poor relevance according to ED OM inside their hospital.

### Phase 2—quantitative methods: framework testing

The results of the qualitative phase inform the following quantitative phase 2 of the research, the purpose of which is to test the framework and identify the most important causes of ED overcrowding.

The ten hospitals in this study offer their availability to provide the data and information needed to calculate the indicators selected in phase 1. In the quantitative part of the research design, we have built a regression model on ED accesses and hospital discharges, using the Principal Components Analysis (PCA) to avoid problems of collinearity among the dependent variables.

Data provided by the ten hospitals were plugged in a regression model in order to estimate the relevance of various groups of variables in explaining patients LOS in EDs. As dependent variable the ED LOS has been chosen since, in scientific literature, it is the most widely used parameter to measure ED overcrowding.

All the variables considered relevant by the experts during the focus groups were included in the model; however, since many of these variables present a high degree of correlation, thus prospecting possible problems related to collinearity, a PCA was used in order to obtain scores which properly summarize the variability within each group. PCA was applied in order to reduce the number of variables to be included in our regression analysis while, at the same time, keeping a proper level of explained variability for each group (we included those main components that explained at least 90% of the overall group variability).

After that, we applied the Shapley Value Decomposition (SVD) to estimate the share of LOS explained variability that could be ascribed to each of the groups previously defined. The SVD is a regression-based decomposition used in cooperative game theory as well as in the analysis of the determinants of inequality. This decomposition is easily employed in any context in which it is necessary to estimate the contribution of a set of variables (considered jointly or as single entities) on a given index (e.g., Gini index, entropy, R^2^). The contribution of any given source of LOS to overall explained variability can be interpreted as the expected marginal impact of the factor when such expectation is made over all possible sequences of elimination. The idea is to specify a regression model to explain LOS by what we consider contributing factors and subsequently decompose the R-squared index isolating the effect of each factor. This methodology presents a significant advantage, because it allows to consider groups of factors as a single entity without affecting their total contribution. Formally we consider the following regression model:

1 $$Y_{ihd}=\beta_0+{\textstyle\sum_{j=1}^n}\;\beta_jG_{ihd}+\epsilon_{ihd}$$

${Y}_{ihd}$ represents LOS for individual *i* admitted in hospital *h* during day *d*. ${G}_{ihd}$ are the *n* groups previously defined in the input-throughput-output model, that include all the scores obtained by the PCA on a set of indicators measuring different dimensions.

The SVD procedures then first calculates the ${R}^{2}$ from the model presented in Eq. (1), which we indicate with ${\widehat{R}}_{tot}^{2}$, then quantifies the factor contributions by removing  ${\widehat{\beta }}_{g}{G}_{ihd}$ and calculates the extent by which ${\widehat{R}}_{tot}^{2}$ changes. The SVD is performed in *n* rounds, equal to the total number of groups. Each round differs according to the number of groups excluded. During the first round, each group is excluded singularly, and the contribution to ${\widehat{R}}_{tot}^{2}$ is obtained as ${{C}_{-j}^{1}= \widehat{R}}_{tot}^{2}-{\widehat{R}}_{-j}^{2}$. During the second round, groups are excluded jointly, two at a time, and their contribution to ${\widehat{R}}_{tot}^{2}$ is obtained as ${{C}_{-j,k}^{2}= \widehat{R}}_{tot}^{2}-{\widehat{R}}_{-j,k}^{2}$; now, ${\widehat{R}}_{-j,k}^{2}$ represents the ${R}^{2}$ value obtained from estimating Eq. (1) after excluding groups *j* ﻿and *k* jointly. We obtain the total contribution to ${R}^{2}$ from group *j* by averaging joint contributions with respect to group *j* as follows: ${C}_{-j}^{2}={\sum }_{k=1}^{n-1}{C}_{-j,k}^{2}$. The subsequent rounds exclude three, four and five groups at a time. In each round, we obtain the contribution to ${\widehat{R}}_{tot}^{2}$ and indicate it as ${C}_{-j}^{n}$. Finally, contributions at each round are averaged across all rounds to obtain the total marginal contribution of each group to ${\widehat{R}}_{tot}^{2}$ as follows: ${C}_{-j}={\sum }_{r=1}^{n}{C}_{-j}^{r}$. In addition, it is possible to obtain Owen values (OVs) that further partition the SV to understand the contribution of each variable in explaining the variability within group j.

### Phase 3—qualitative methods: contribution to the interpretation of the quantitative findings

Finally, the results of the statistical analysis are discussed in a final focus group with participants, who are summoned with the purpose of contributing in the identification of possible OM strategies to be adopted in the hospital to optimize ED patient flow.

## Findings

### Operational framework

After the iterative process conducted during the focus groups with the ten hospitals included in the study, a list of variables to be included in the framework for the evaluation of hospital ED operations management is identified (see Table 3).

**Table 3:** Indicators used in the model

Indicator	Variable Meaning	Source
**ED Input**		
Share of vulnerable population (age > = 75 years) per day	Elderly individuals need more intensive services, and consequently, a higher share of patients over 75 years of age should be correlated with higher ED	ED database
Share of red codes above average per day^a^	Red codes require immediate attention from all ED team members	ED database
Number of admissions	This represents the workload level in the ED	ED database
Share of red and yellow codes above average per day^a^	This is the daily share of major codes (red and yellow) that require more intensive care	ED database
**ED Process**		
ED endowment		
ED type	This represents, in a three-scale variable, the endowment of technology and diagnostic equipment of each single hospital	Semistructured interview
Number of doctors per admission	This represents the quantitative level of medical staff	Semistructured interview
Number of nurses per admission	This represents the quantitative level of nursing staff	Semistructured interview
Skill mix (number of doctors/number of nurses)	This represents the type of organizational model in terms of skill mix for nurses vs. physicians	Semistructured interview
Visual management software	This is the availability of real time information on hospital bed occupancy	Semistructured interview
ED flow separation		
Ambulatory for minor codes^a^	This refers to the outpatient setting managed by primary care doctors dedicated to white codes	Semistructured interview
See and treat	This refers to the outpatient setting managed by nurses for the treatment of minor pathologies	Semistructured interview
Fast track	This refers to direct access to outpatient specialties for specific clinical conditions	Semistructured interview
**ED Output**		
ED hospital admissions	This is the number of patients who require hospital admission after ED visits	Hospital discharge database
ED hospital admissions/Medicine ward	This is the number of patients admitted to the hospital in the medical ward from the ED	Hospital discharge database
ED hospital admissions/Emergency ward	This is the number of patients admitted to the hospital in the emergency medicine ward from the ED	Hospital discharge database
ED hospital admissions/Surgery ward	This is the number of patients admitted to the hospital in the surgical ward from the ED	Hospital discharge database
“In and out” rate	This is the daily difference between the number of admissions and of discharges	Hospital discharge database
Bed manager	This refers to the availability of a team in charge of coordinating patient flows between the ED and hospital wards	Semistructured interview
**Hospital**		
Hospital ownership	This can be private vs. public	Semistructured interview
Geographical context	This can be urban vs. rural	Semistructured interview
Hospital dimension	This can be the number of beds	Semistructured interview
Hospital case mix of production	This can be the number of medical vs. surgical patients	Hospital discharge database
Hospital organization model	This can be process- vs. specialty-based hospitals	Semistructured interview
Surgical capacity	This refers to the number of operating rooms	Semistructured interview

^a^In Italy, at the check-in point, patients are evaluated by nurses who assign them a colour according to their health needs. There are four level of severity: (i) red means life-threating conditions, (ii) yellow stands for potentially life-threatening conditions, (iii) green denotes minor injuries or illnesses, and (iv) white stands for nonurgent conditions

For each variable, the information and data needed to calculate the corresponding indicators are detailed according to three different sources: the administrative hospital database (i.e., the hospital discharge database), the ED administrative hospital database, and information on the ED organizational model.

Specifically, hospitals provide two types of data: (i) hospital discharge data, which trace information about activities and procedures delivered to the patient within the hospital, and (ii) emergency department data, which trace information about the different timings of the patient within the ED from his or her very arrival to his or her final discharge in another setting that can be, alternatively, the patient’s home, hospital ward, outpatient department or another institution. A formal request (available upon request) is sent to the ten hospitals included in the study specifying, for each of the two databases, the exact variables needed and the procedures used to respect patients’ privacy. Data cover the period from January 1st to December 31st, 2018.

To collect the information on the ED organization model, a semistructured interview (available upon request) is administered to the teams of the ten hospitals, aiming to collect information on five different thematic areas:

1. description of patient flow logistics in the ED,
2. admission process of ED patients to other hospital settings through the ED,
3. role and procedures for bed management,
4. ED personnel, and
5. information and communication technology (ICT) system adopted to support ED management.

To evaluate whether other hospital variables may influence ED performance, some variables are included in the model to measure general hospital characteristics: i) type of ownership (private vs. public), ii) geographical context (urban vs. rural), iii) number of beds, iv) production case mix (medical vs. surgical patients), v) type of organization model (process- vs. specialty-based hospital), and vi) number of operating rooms.

### Regression analysis

Table 4 summarizes the main findings of the model for three different samples:

1. Subsample #1, which excluded patients who have been through the observational unit (NO OU model, *n* = 560,178),
2. Subsample #2, which includes only cases admitted to the hospital (ADM model, *n* = 88,361), and
3. Subsample #3, which includes patients who have not been admitted to inpatient facilities and have not been through an observational unit (NO OU – NO ADM model, *n* = 488,098).

**Table 4:** Main results of the regression model

		**Sample #1** **NO OU model**			**Sample #2** **ADM model**			**Sample #3** **No OU—No ADM model**		
			**Owen**	**Shapley**		**Owen**	**Shapley**		**Owen**	**Shapley**
**Context**	pc1 (bed endowment)	-0,019	5,71	**8,66**	-0.084*	2,09	**8,13**	-0,03	3,91	**7,21**
		(0.0166)			(-0.033)			(0.0168)		
	pc2 (intensity of care)	0.076***	1,58		0.514***	2,81		0.064***	1,78	
		(0.0132)			(0.0513)			(0.0105)		
	pc3 (% of medical patients)	-0.127**	1,37		-0.569***	3,23		-0.160***	1,52	
		(0.0389)			(0.0778)			(-0.04)		
**Input**	pc1 (admissions)	0.054***	6,73	**16,46**	0.055***	2,56	**3,92**	0.061***	13,51	**26,21**
		(0.0073)			(0.0149)			(0.0074)		
	pc2 (case mix)	0.016**	6,71		0.031**	1,03		0.018***	7,85	
		(0.0047)			(0.0103)			(0.0049)		
	pc3 (elderly individuals)	0.023***	3,02		0.033***	0,33		0.026***	4,85	
		(0.0037)			(0.0082)			(0.0038)		
**Process (ED endowment)**	pc1 (skill mix)	0.165***	2,96	**30,63**	0.678***	6,15	**13,57**	0.186***	5,17	**37,74**
		(0.0275)			(0.0669)			(0.0281)		
	pc2 (# of nurses)	-0.032**	21,38		0,023	3,15		-0.057***	26,98	
		(-0.011)			(0.0223)			(0.0111)		
	pc3 (ED endowment)	-0,052	6,29		-0,155	4,27		-0.204***	5,59	
		(0.0452)			(0.0792)			(0.0462)		
**Process (ED flow separation)**	pc1 (minor codes)	0.104***	1,42	**2,41**	0.489***	3,74	**7,1**	0.184***	3,24	**4,64**
		(0.0145)			(0.0372)			(0.0153)		
	pc2 (fast track/see and treat)	0.160***	0,99		0.446***	3,36		0.321***	1,4	
		(0.0279)			(0.0613)			(0.0311)		
**Output: Admissions management**	pc1 (hospital admissions)	0.079***	15,77	**30,31**	0.261***	9,88	**60,43**	0.051***	12,41	**15,94**
		(-0.004)			(0.0092)			(0.0037)		
	pc2 (medical patients)	-0.011***	2,47		0.169***	4,56		0.061***	3,52	
		(0.0026)			(0.0108)			(0.0043)		
	pc3 (surgical patients)	0.041***	0,95		0.314***	7,2		-	-	
		(0.0034)			(0.0081)			-		
	pc4 (bed manager)	0.101***	10,34		-0.748***	35,62		-	-	
		(0.0037)			(0.0159)			-		
	pc5 (out/in)	-0.029***	0,79		-0.070***	3,17		-	-	
		(0.0031)			(0.0107)			-		
**Output: Emergency path**	pc1 (emergency wards)	-0.107***	2,37	**11,53**	-0.457***	3,93	**6,84**	-0.084***	1,53	**8,27**
		(-0.016)			(-0.034)			(0.0165)		
	pc2 (OR dedicated to the ED)	0.133***	9,16		0,026	2,91		0.135***	6,74	
	**R**^**2**^	**4%**			**15%**			**3%**		
	N	560.178			88.361			488,908		
				**100.00**			**100.00**			**100.00**

**Notes:** Standard errors are clustered at the hospital/day level. Significance levels:

^***^p < 0.01

^**^p < 0.05, and

^*^p < 0.1

The aim of running the model on these three different subsamples is as follows:

1. not including the time spent in the OU in the ED length of stay; in fact, the aim of the OU is to study/observe the patient conditions and to decide, after a specific length of time (minimum of 6 h and up to 36 h), whether to admit him or her to the wards (or send him or her back home or to other external facilities), and
2. separate analysis for ED patients who have been admitted to the hospital’s wards and those who have been discharged in other settings (including patient home).

The statistical model clearly shows that some dimensions do have a relevant and significant impact on overall ED length of stay. As explained in the methodology section, to avoid possible problems of collinearity, we group those factors that actually represent a single phenomenon.

Table 4 summarizes the main results of the model for the three different subsamples analysed. In particular, they present three different pieces of information: (i) ordinary least squares (OLS) estimates for the coefficients associated with PCA; (ii) Owen values (OVs), defined in the session dedicated to the methodology; and (iii) the SVs for each group considered.

If we focus on PCA scores obtained from context-related indicators, we can see that coefficients associated with the second and third components are significant across the samples analysed. These components, according to the results presented in Table 4, can be considered proxies for ED intensity of care (pc2) and the percentage of medical patients (pc3), and in line with our expectations, the former (latter) is positively (negatively) correlated with ED LOS.

Considering the PCA scores obtained from input-related indicators, we can see that the coefficients associated with all components are significant across the samples analysed. Again, referring to Table 4, we interpret these components as proxies for admissions (pc1), case mix (pc2) and elderly patients (pc3). These components are positively correlated with ED LOS.

Considering the PCA scores obtained from ED endowment-related indicators (process dimension), we can see that coefficients associated with the first two components are significantly different from zero in Subsample #1, the coefficient of the second component is significantly different from zero in Subsample #2, and all coefficients are significant in Subsample #3. We interpret these components as proxies for skill mix (pc1), number of nurses (pc2) and ED endowment (pc3). Considering Subsamples #1 and #3, we find a positive (negative) correlation between ED LOS and skill mix (number of nurses). In addition, in Subsample #3, ED endowment is negatively correlated with ED LOS. Finally, in Subsample #2, only skill mix is positively correlates with ED LOS.

Considering the PCA scores obtained from ED flow separation-related indicators (process dimension), we can see that coefficients associated with all components are significant across the samples analysed. We interpret these components as proxies for minor codes (pc1) and the presence of fast-track/see-and-treat procedures (pc2). These components are positively correlated with ED LOS across each sample analysed.

Considering the PCA scores obtained from admission management-related indicators, we can see that the coefficients associated with all components are significant across the samples analysed. We interpret these components as proxies for hospital admissions (pc1), medical patients (pc2), surgical patients (pc3), bed managers (pc4) and out/in (pc5). In line with our expectations, we find a positive influence of hospital admissions, whereas we find evidence of a negative correlation between surgical patients (pc3) and bed managers in terms of ED LOS and medical patients (pc2) and out/in across Subsamples #1 and #2.

Finally, considering the PCA scores obtained from emergency-related indicators (output dimension), we outline the strong negative association with pc1 that represents the presence of an emergency ward.

If we focus on the Shapley values, we can see that across the three different subsamples, the most important group of variables is that considering the output dimension connected to admission management; the share of explained variability, in fact, amounts to 30.31%, 60.43% and 15.94% for Subsamples #1 (NO OU model), #2 (ADM model) and #3 (NO OU – NO ADM model), respectively.

When looking at the most important PCA scores across subsamples, we can confirm the relevance of hospital admissions across all the samples analysed (15.77%, 9.88% and 12.41% for Subsamples #1, #2, and #3, respectively) and the presence of a bed manager in Subsamples #1 and #2 (10.34% and 35.62%, respectively).

According to the SVD analysis, the second most relevant group of variables in explaining ED LOS is represented by the process dimension connected with ED endowment, which explains 13.05% of overall variability. This dimension is also relevant for each of the subsamples analysed, where the share of explained variability amounts to 30.63%, 13.57% and 37.74% for Subsamples #1 (all patients except those who have passed through the OU), #2 (ED patients admitted to hospital) and #3 (ED patients who have not gone through the OU and have not been admitted to the hospital), respectively.

Thus, the variable ED endowment is particularly relevant only for Subsample #3, suggesting that the availability of technology and equipment is not significant for more urgent and complex cases.

Within this dimension, the PCA scores correlated with the number of nurses are those that explain most of the variability in the overall population and in other subsamples, except that for Subsample #2 (those patients who require admission).

The third most relevant group of variables in explaining ED LOS is represented by the input dimension, which explains 9.96% of overall variability. This dimension is also relevant for Subsamples #1 and #3, where the share of explained variability amounts to 16.46% and 26.21%, respectively. Within this dimension, the PCA scores correlated with admissions, case mix and presence of elderly patients are all almost always equally relevant.

The three dimensions discussed above (input, process and output) explain, for each of the subsamples considered, 77%, 78% and 80% of overall ED LOS variability. The other groups of variables considered have a more limited relevance. In particular, the presence of separated pathways for minor codes does not play a relevant role. This result is probably due to two different factors: (i) it is a dichotomous variable (Yes/No), and (ii) most of the hospitals included in the sample have implemented this type of organizational model.

Finally, in all samples, the hospital dimension plays a marginal role in explaining the dependent variable.

## Discussion

The findings of this study summarized in the previous paragraph help the authors accomplish two different goals: (i) the operationalization of the input-process-output model through the creation of a balanced dashboard of indicators that are useful for measuring the performance of emergency patient flow management, which can be adopted across sites and times, and (ii) providing managers and policy makers with an evidence-based road map for the redesign of ED patient flows capable of considering all the interdependencies between ED and all other hospital production units.

The use of a three-step mixed-method approach helped to strengthen the relevance of the results. The semistructured interviews helped gathering information about some organizational variables (e.g. the presence of a beds management function) that turned to be statistically relevant in explaining ED LOS. The results of our logistical model show clear and robust trends that offer both managers and policy makers powerful hints for redesigning ED operations in all three main components: (i) input, (ii) process and (iii) output. Finally, during the final focus group the participants discussed these results with the purpose of contributing in identifying possible OM strategies to be adopted in the hospital to optimize ED patient flow.

Table 5 summarizes the most relevant insights of this study, which paves the way for possible future scenarios in the redesigning of ED patient flow management.

**Table 5:** Most relevant explanatory variables of ED crowding

Variable	Findings	Policy and managerial implications
**Input**		
Share of vulnerable population (age > = 75 years) per day	The study confirms the evidence found in other studies that patient characteristics do have an impact on ED crowding. The study proves also that the variability of ED arrivals does have an impact on ED operations.	Separate ED patient flows based on scores that consider different patient characteristics such as age, severity, comorbidities.
Number of admissions		Work on scheduling and capacity planning to better match demand and supply in the busiest periods.
**Process**		
Number of nurses per admission	The paper confirms the results of other studies that sustain the presence of an ED capacity problem. In particular, the model stresses the relevance of nurse shortages.	Hire more nurses. Improve solutions enabling the saving of nursing time.
Skill mix	ED crowding is found to be positively correlated with the physician-to-nurse ratio	Tailor solutions to the specific ED context, mission and goals.
**Output**		
Number of ED hospital admissions	The study shows the statistical relevance of specific variables that better operationalize the coordination between ED and bed management concerning the ward issue.	Streamline the discharge process: (i) discharge room or (ii) re-engineering wards’ operations.
“In and out” rate		
Bed manager		Set up of an office in charge of coordinating bed management.
ED hospital admissions/Emergency ward		Set-up an emergency ward or an admissions unit as a buffer area between the ED and hospital wards.

Regarding input, our study confirms a recent trend in the literature that outlines that ED crowding is significantly influenced by the typology of patients, such as age, clinical complexity, presence of comorbidities or level of urgency. In particular, our study proves that crowding worsens as the mean age of patients in the ED increases.

Furthermore, it has been proven that ED performance is also influenced by the trend in the number of arrivals, an aspect not typically considered in the recent literature on ED crowding. In other words, the probability of waiting is significantly higher during those hours or days where we record a higher number of arrivals.

Looking at ED processes, in the cases analysed, there is, for sure, a capacity problem; the model, in particular, shows that the number of nurses assigned to each single shift has a relevant impact on ED crowding.

Another interesting finding is represented, in contrast, by the data of the positive correlation between ED LOS and the physician-to-nurse ratio. This circumstance seems to suggest that those EDs where there is a higher presence of doctors tend to anticipate therapeutic and diagnostic activities in the ED, which in turn increases overall length of stay. This consideration opens up a broader discussion about the role that an ED should play in the overall hospital production system: (i) either the mere stabilization of the patient and sending him or her to hospital wards or (ii) the management of the initial phase of the diagnostic and medical treatment of the patient’s pathway.

Finally, looking at the output, our results are consistent with those of other studies that show how ED problems are not only ED problems; in contrast, such problems represent a broader hospital issue linked to the balance between the number of hospital admissions and discharges. Our model, in particular, outlines the statistical relevance of the following variables: (i) the number of hospital admissions (e.g., number of daily hospital admissions from the ED); (ii) the daily difference in the number of discharges and hospital admissions; (iii) the presence, within the hospital, of a bed management function; and (iv) the presence of an emergency ward to accommodate patients admitted to the hospital through the ED.

### Policy and managerial implications

The present study, combining qualitative and statistical evidence, provides robust evidence to allow for the sketching of an evidence-based road map for the redesign of ED patient flow logistics around the three broad dimensions considered.

Looking at the input component of the model, our study confirms the evidence stemming from a growing body of scientific literature that patients’ characteristics do have an impact on ED crowding. This element calls for the implementation of the well-known operational principles of flow separation through the creation of dedicated pathways for fragile, complex and elderly patients. This scenario requires the creation of appropriate scores that are capable of detecting these patients from their very first access to the ED.

Furthermore, the present study supports a quite intuitive circumstance: ED crowding is significantly influenced by the daily variability of patient arrivals. This variability is a typical natural variability, that is, a variability that cannot be eliminated but only more efficiently managed through, for example, a scheduling system that allocates nurses and physicians in a more productive manner. We know, for example, by looking at our data and the findings of other studies [33], that the peak of the number of arrivals within the day occurs in the early morning between 8 a.m. and 10 a.m. Over the course of the week, Monday is typically the busiest day. To better manage such peaks, it is possible to work on another important logistical driver—capacity planning—to increase the number of physicians and nurses or strengthen supportive services such as diagnostics and social services during these peak times.

For the process component, the novel and interesting result about the relevance of skill mix proves the fact that one-size-fits-all solutions do not exist; in contrast, solutions need to be shaped around the specific role and mission of each ED. In those EDs characterized by higher physician-to-nurse ratios and more inclined to anticipate medical treatment before admission to the ward, the overall length of stay is higher.

This study also proves that part of ED crowding is linked to a problem of capacity; in particular, the model shows the relevance of the variable “number of nurses per shift”. There are two main strategies through which to overcome this problem: (i) hiring more nurses (solution that has an economic impact) or (ii) adopting organizational and logistical solutions to save nursing time through, for example, shifting some typically nursing tasks to other roles such as scribes or social workers.

Finally, as for the output component, the study provides some clear indications about the possible strategies through which to solve the coordination problems between the ED and hospital wards: (i) create an emergency ward or an admissions unit as a buffer area between the ED and hospital wards; (ii) set-up an office in charge of coordinating bed management (possibly included within a hospitalwide operations management function) with responsibilities such as the assessment of bed availability in real time and the triage and admission of patients; and (iii) streamline the discharge process through, for example, a different organization of the activities in the ward or the constitution of a discharge room to alleviate the problem of a lack of beds, especially during the morning hours when the pressure from the ED is more intense.

This study, performed just before the surge of the current COVID-19 pandemic, has shown that Italian EDs were operating at maximum capacity and that they were highly exposed to variability in demand in terms of both the gross number of arrivals and the level of severity of coming patients. This circumstance has turned out to be dramatically true during the current pandemic, where, in many areas of the country, EDs have not been capable of dealing with the surge in the number of arrivals due to the pandemic.

The hope is that once the pandemic is over, healthcare managers and policy makers will be granted the resources, time, lucidity and foresight to redesign ED operations for the long term along the lines of the evidence-based suggestions indicated by the results discussed in this work.

As previously stated, the present study focuses on internal hospital operations with the goal of identifying a balanced dashboard to control ED patient flow logistics and defining a possible coherent plan for change. Future research and efforts should focus on the coordination of hospital operations with other production units upstream (e.g., primary care doctors or nursing homes) and downstream (e.g., intermediate care or rehabilitation centres).

## Data Availability

The datasets generated during the current study are not publicly available due to confidentiality of some of the data but are available from the corresponding author upon reasonable request.

## Abbreviations

ED: Emergency Department
LHA: Local Health Authorities
LOS: Length of Stay
NHS: National Health Service
OLS: Ordinary Least Squares
OU: Observational Unit
OV: Owen Values
PCA: Principal Component Analysis
SVD: Shapley Value Decomposition
